# Low Concentrations of *o,p’*-DDT Inhibit Gene Expression and Prostaglandin Synthesis by Estrogen Receptor-Independent Mechanism in Rat Ovarian Cells

**DOI:** 10.1371/journal.pone.0049916

**Published:** 2012-11-27

**Authors:** Jing Liu, Meirong Zhao, Shulin Zhuang, Yan Yang, Ye Yang, Weiping Liu

**Affiliations:** 1 MOE Key Lab of Environmental Remediation and Ecosystem Health, College of Environmental and Resource Sciences, Zhejiang University, Hangzhou, China; 2 Research Center of Environmental Science, College of Biological and Environmental Engineering, Zhejiang University of Technology, Hangzhou, China; Baylor College of Medicine, United States of America

## Abstract

*o,p’*-DDT is an infamous xenoestrogen as well as a ubiquitous and persistent pollutant. Biomonitoring studies show that women have been internally exposed to *o,p’*-DDT at range of 0.3–500 ng/g (8.46×10^−10^ M−1.41×10^−6^ M) in blood and other tissues. However, very limited studies have investigated the biological effects and mechanism(s) of *o,p’*-DDT at levels equal to or lower than current exposure levels in human. In this study, using primary cultures of rat ovarian granulosa cells, we determined that very low doses of *o,p’*-DDT (10^−12^−10^−8^ M) suppressed the expression of ovarian genes and production of prostaglandin E2 (PGE2). *In vivo* experiments consistently demonstrated that *o,p’*-DDT at 0.5–1 mg/kg inhibited the gene expression and PGE2 levels in rat ovary. The surprising results from the receptor inhibitors studies showed that these inhibitory effects were exerted independently of either classical estrogen receptors (ERs) or G protein-coupled receptor 30 (GPR30). Instead, *o,p’*-DDT altered gene expression or hormone action via inhibiting the activation of protein kinase A (PKA), rather than protein kinase C (PKC). We further revealed that *o,p’*-DDT directly interfered with the PKA catalytic subunit. Our novel findings support the hypothesis that exposure to low concentrations of *o,p’*-DDT alters gene expression and hormone synthesis through signaling mediators beyond receptor binding, and imply that the current exposure levels of *o,p’*-DDT observed in the population likely poses a health risk to female reproduction.

## Introduction

Numerous studies have proposed the ever-disturbing results of endocrine disrupting chemicals (EDCs) as culprits in the decline in male and female fertility potential [1]. Dichlorodiphenyltrichloroethane (DDT), a widely used pesticide as well as a ubiquitous and persistent pollutant, is an infamous EDC [1]. Although use of DDT has been banned in most industrialized nations for 30 years, in some developing countries where DDT is still sprayed for malaria control, the general population is exposed to particularly high DDT levels [2]; [3]. Even in areas where DDT has been restricted to use, residues of DDT concentrations continue to be reported worldwide in environment and biota because of its long half-life [4]; [5]. Noticeably, DDT is consistently found in serum and ovarian follicular fluids of women [6]–[8], which may associate with adverse impacts on female fertility, menstrual cycles, and time to pregnancy [9]–[12]. Thus, there continues to be ongoing concern over the risk to human reproductive health of DDT at current environmental exposure levels. However, its risk to human is not easily answered because of the contradictory epidemiologic results regarding the controversial relationships of serum DDT levels and human reproductive disorders [13]; [14]. Therefore, we must depend on *in vitro* and *in vivo* studies as a guide to determine rational safety levels for humans.

The technical mixtures of DDT contain two major components, *p,p’*-DDT (77%) and *o,p’*-DDT (15%–23%). The *o,p’*- isomer is the most estrogenic component of technical DDT [15]; [16]. The binding ability of *o,p’*-DDT to estrogen receptors (ERs) is 100-fold greater than that of *p*,*p*’*-*DDT [15]. The animal experiments indicated that human blood concentrations of *o,p’*-DDT could reach estrogenic level [17]. Biomonitoring studies show that women have been internally exposed to this potent xenoestrogen at range of 0.3–500 ng/g (8.46×10^−10^ M−1.41×10^−6^ M) in blood and other tissues [3]; [18]–[22]. Previous evidence has demonstrated the deleterious effects of *o,p’*-DDT at high doses on reproductive tissues [23]; [24]. However, very limited studies have investigated the biological effects of *o,p’*-DDT at levels equal to or lower than current exposure levels in human. It has been reported that *o,p’*-DDT at concentration of 4 ng/ml inhibited the synthesis of estrodial (E_2_) and progesterone in ovarian granulosa cells [25]. However, the exact mechanism of the action of *o,p’*-DDT at low environmental doses remains amphibolous.

In the present study, we utilized primary cultured rat granulosa cell as an experimental model, as this cell is a well accepted *in vitro* model to test the toxic potential of EDCs on female reproduction [26]–[28]. We determined the *in vitro* effects of *o,p’*-DDT at low doses (10^−12^−10^−8^ M) on ovarian gene expression and prostaglandin synthesis. We further investigated whether *o,p’*-DDT alters the gene expression via the estrogen receptor (ER) pathway, as well as protein kinase A (PKA) and protein kinase C (PKC), which are critical signaling mediators for ovarian gene expression and prostaglandin synthesis. Moreover, we assessed the *in vivo* impacts of *o,p’*-DDT at doses of 0.1–1 mg/kg on the expression profiles of ovarian genes, prostaglandin synthesis and activities of protein kinases.

**Table 1:** Primer sequences for Real-time PCR.

Gene name	Primer sequences	
	Forward	Reverse
L32	5′-TGGTCCACAATGTCAAGG-3′	5′-CAAAACAGGCACACAAGC-3′
P450scc	5′-CTATGCCATGGGTCGAGAAT-3′	5′-CAGCACGTTGATGAGGAAGA-3′
StAR	5′-ACATATGCGGAACATGAAAGG-3′	5′-GCTGGATGTAGGACAGCTCC-3′
PR	5′-CCAGAGCCCACAATATGG-3′	5′-TAAATAGTTATGCTGCCCTTCC-3′
SULT1E1	5′-GATGAAGAACAATCCATGCACC-3′	5′-CTCCTCAAATCTCTCCCTCAGG-3′
COX-2	5′-GATCACATTTGATTGACAGC-3′	5′-TCCTTATTTCCTTTCACACC-3′
EREG	5′-TATCAGCACAACCGTGATTCC-3′	5′-ATGCAAGCAGTAGCCGTCC-3′
RUNX1	5′-AACCCTCAGCCTCAAAGTCA-3′	5′-GGGTGCACAGAAGAGGTGAT-3′
p21	5′-ACCCCTGTTTCTGTAACACC-3′	5′-GAAGTATTTATTGAGCACCAGC-3′

## Materials and Methods

### Materials

The pesticide (*o,p’*-DDT) and ER inhibitors (ICI 182780 and G15) were purchased from Sigma (St. Louis, MO, USA). Pregnant mare serum gonadotropin (PMSG), human chorionic gonadotropin (hCG) were purchased from ProSpec-Tany TechnoGene Ltd. (East Brunswick, NJ, USA).

**Figure 1:**
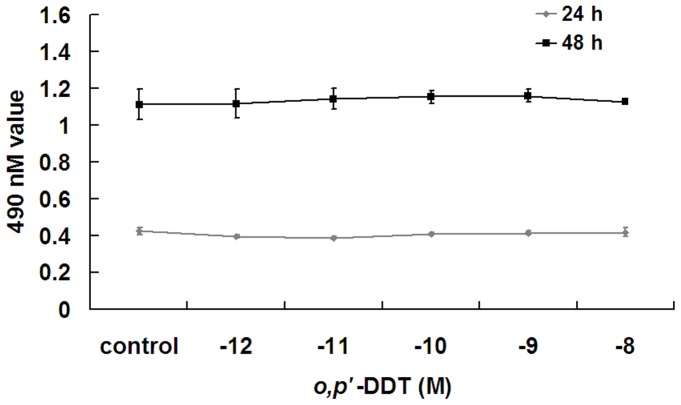
Low concentrations of *o,p’*-DDT have no effects on cell viability of rat ovarian granulosa cells. Granulosa cells were exposed to *o,p’*-DDT (10^−12^ to 10^−8^ M) for 24 h or 48 h and cell viability was measured using MTS assay.

### Animals and Culture of Rat Ovarian Granulosa Cells

Sprague Dawley female rats were obtained from Shanghai Laboratory Animal Center, Chinese Academy of Sciences (Shanghai, China). All animal procedures were approved by the Zhejiang University Animal Care and Use Committee. Female rats reach their sexual maturity at about 5 weeks of age and they have the endogenous follicle-stimulating hormone (FSH) and luteinizing hormone (LH) surge at this age. These gonadotropin surges initiate the growth of ovarian granulosa cells, the expression of ovulatory genes and the synthesis of ovarian prostaglandins. To avoid the interference of endogenous gonadotropins on experimental results, we used sexually immature rats on PND 21–26 before they have the first endogenous gonadotropin surge [28]–[31]. The immature rats are injected with FSH agonist, PMSG (10 IU) to mimic FSH action that stimulates granulosa cell growth, and subsequently treated with LH agonist, hCG to mimic LH surge that induces gene expression and prostaglandin synthesis.

**Figure 2:**
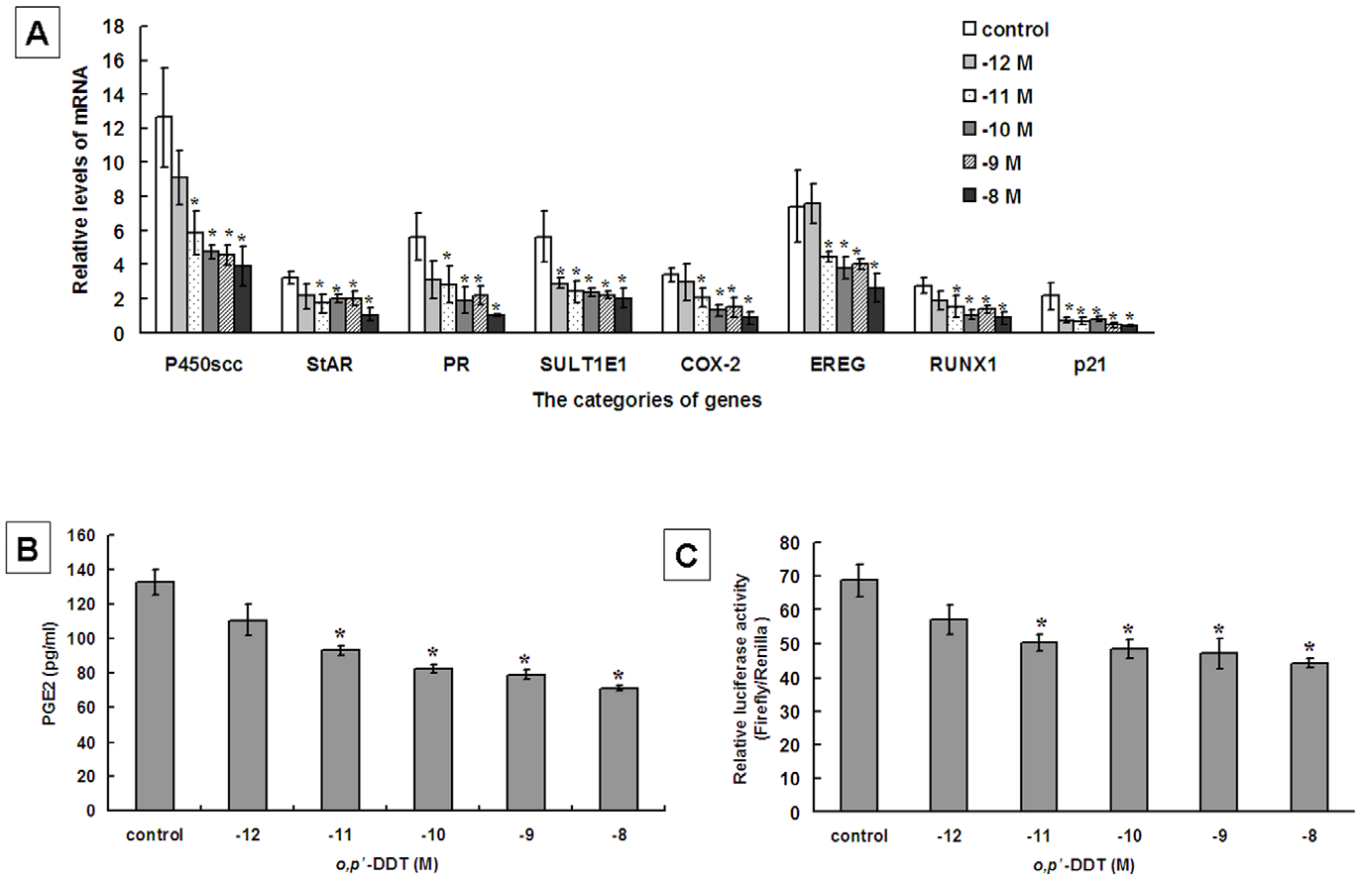
Low concentrations of *o,p’*-DDT inhibit *in vitro* gene expression, PGE2 secretion and transcriptional activity of COX-2 promoter in primary cultures of rat ovarian granulosa cells. (**A**) *In vitro* expression levels of mRNA for ovarian genes in granulsa cells exposed to *o,p’*-DDT at concentrations of 10^−12^−10^−8^ M. (**B**) Levels of PGE2 in cultured media of rat granulosa cells exposed to *o,p’*-DDT at concentrations of 10^−12^−10^−8^ M. (**C**) Luciferase activity of COX-2 promoter in granulsa cells exposed to *o,p’*-DDT at concentrations of 10^−12^−10^−8^ M. The results were shown as mean ± SEM for three independent experiments performed in triplicate. *, *ρ* <0.05, compared to control.

For *in vitro* studies, animals were scarified at 48 h after PMSG administration and ovaries were collected to isolate and culture granulosa cells as described previously [29]; [30]. Cells were cultured in HyQ MEM-RS (ThermoFisher Scientific, Waltham, MA, USA) media supplemented with 0.05 mg/ml gentamicin and 1× insulin, transferrin, and selenium, as well as with 1 I U/ml hCG to mimic LH action that induces ovarian gene expression and prostaglandins production *in vitro* [32]; [33]. Cells were exposed to *o,p’*-DDT at concentration of 10^−12^ to 10^−8^ M for 6 h. For inhibitors experiments, cells were pretreated with inhibitors for 30 min and subsequently treated with the combination of *o,p’*-DDT and inhibitor. Because *o,p’*-DDT was dissolved in ethanol for experiments, the same concentration of ethanol was added to medium for the control cells. The final concentration of ethanol was 0.1% (vol/vol).

**Figure 3:**
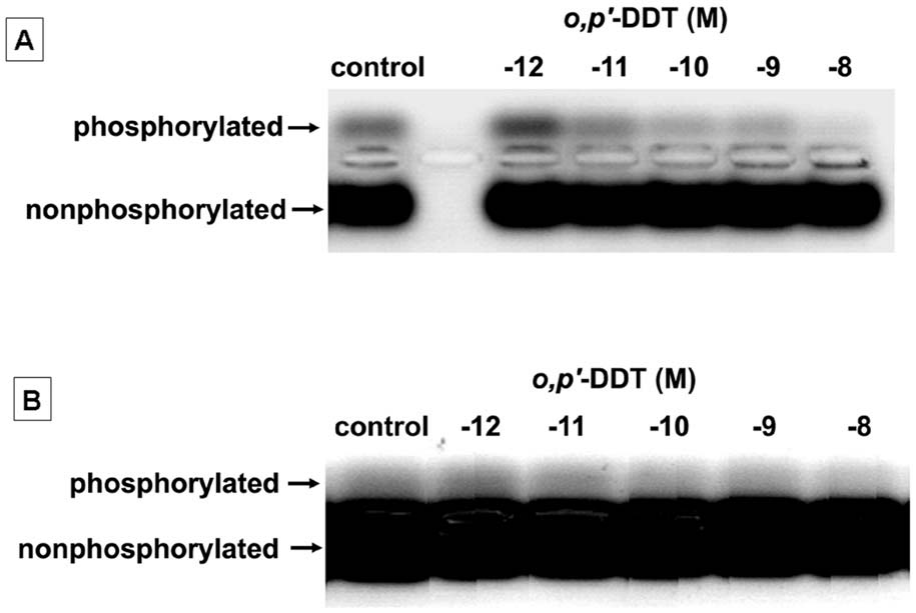
Low concentrations of *o,p’*-DDT inhibit PKA but not PKC activity in primary cultures of rat ovarian granulosa cells. (**A**) PKA activity (phosphorylated band) in granulosa cells exposed to *o,p’*-DDT at concentrations of 10^−12^−10^−8^ M. (**B**) PKC activity (phosphorylated band) in granulosa cells exposed to *o,p’*-DDT at concentrations of 10^−12^−10^−8^ M. The data represented similar results from three independent experiments.

For *in vivo* studies, immature female rats (21-day old) received daily intraperitoneal (i.p.) injections of *o,p’*-DDT in corn oil: 0.1 mg/kg, 0.5 mg/kg, 1 mg/kg, or corn oil alone (control). Animals were injected with PMSG (10 IU) s.c. at the age of 24-day. Forty-eight hours later, rats were injected hCG (10 IU) s.c. to induce the production of prostaglandins and ovulatory genes expression**.** Control and exposed animals were sacrificed at 6 h after hCG administration and ovaries were collected to isolate the granulosa cells.

**Figure 4:**
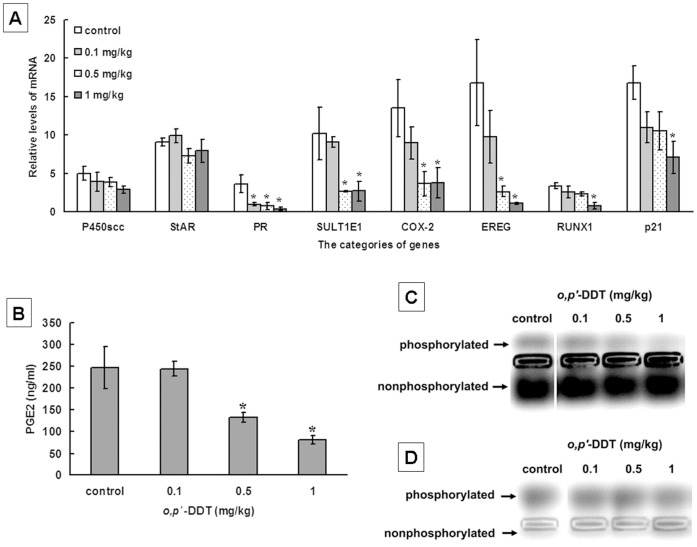
Low doses of *o,p’*-DDT affect the *in vivo* expression of genes, PGE2 secretion and PKA activity in rat ovaries. Female rats were received daily i.p. injections of *o,p’*-DDT at doses of 0.1, 0.5, 1 mg/kg/day for 6 days. Ovaries were collected to isolate the granulosa cells. (**A**) *In vivo* expression levels of mRNA for ovarian genes. (**B**) Levels of PGE2 in rat ovarian tissues. (**C**) *In vivo* PKA activity (phosphorylated band) in rat ovarian tissues. (**D**) *In vivo* PKC activity (phosphorylated band) in rat ovarian tissues. The results of (**A**) and (**B**) were shown as mean ± SEM for three independent experiments performed in triplicate. The data of (**C**) and (**D**) represented similar results from three independent experiments. *, *ρ* <0.05, compared to control.

### MTS Cell Viability Assay

Granulosa cells were seeded in 96-well plates (5000 cells per well) and exposed to *o,p’*-DDT (10^−12^ to 10^−8^ M) for 24 h or 48 h. Cell viability was measured using CellTiter 96 AQueous One Solution Cell Proliferation (MTS assay) (Promega) according to the manufacturer’s protocol. Briefly, at end of culture, 20 µl of reagent were pipetted into each well containing the cells in 100 µl of culture medium, and cells were then returned to the incubator for an additional 2 h. The absorbance was measured at 490 nm in the Infinite M200 plate reader (Tecan USA, Durham, NC, USA) to determine the formazan concentration, which is proportional to the number of live cells.

**Figure 5:**
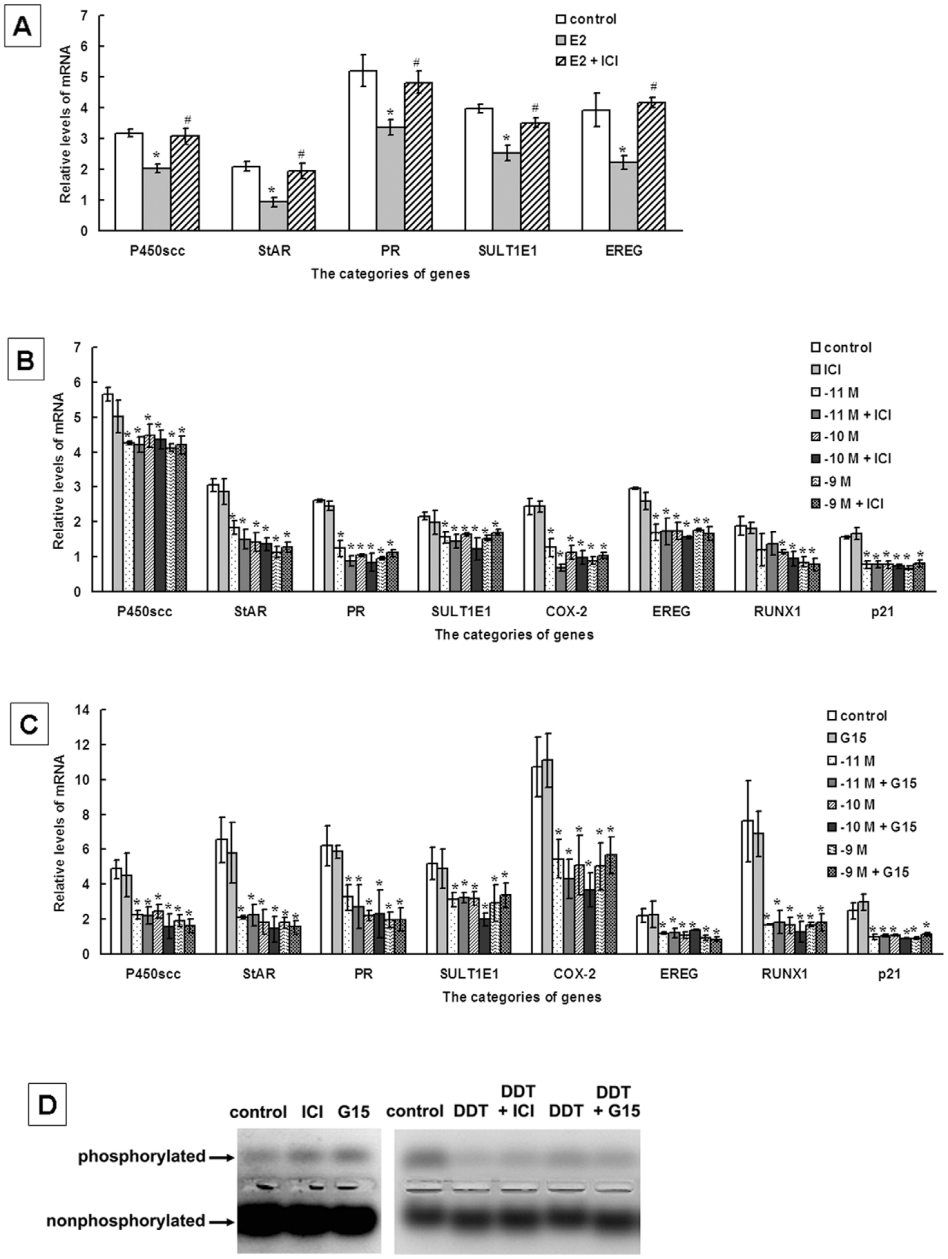
*o,p’*-DDT represses the expression of ovarian genes and PKA activity by ERs- and GPR30-independent pathway. (**A**) Levels of mRNA for ovarian genes in rat ovarian granulosa cells exposed to 10^−10^ M E_2_ with or without the pretreatment of 10^−7^ M ICI 182780 (ICI), specific inhibitor for ERs (α and β). (**B**) Levels of mRNA for ovarian genes in granulosa cells exposed to 10^−11^−10^−9^ M *o,p’*-DDT with or without the pretreatment of 10^−7^ M ICI 182780 (ICI). (**C**) Levels of mRNA for ovarian genes in granulosa cells exposed to 10^−11^−10^−9^ M *o,p’*-DDT with or without the pretreatment of 10^−7^ M G15, specific inhibitor for GPR30. The results of three independent experiments performed in triplicate were shown as mean ± SEM. (**D**) PKA activity (phosphorylated band) in rat granulosa cells exposed to 10^−10^ M *o,p’*-DDT with or without the pretreatment of 10^−7^ M ICI 182780 (ICI) or G15. The results of (**A**), (**B**) (**C**) and were shown as mean ± SEM for three independent experiments performed in triplicate. The data of (**D**) represented similar results from three independent experiments. *, *ρ* <0.05, compared to control; #, *ρ* <0.05, compared to E_2_ treatment.

### Quantification of mRNA

Total RNA was isolated from granulose cells using Trizol reagent according to the manufacturer’s protocol (Invitrogen). Synthesis of first-strand cDNA was performed by reverse transcription of 1.0 µg total RNA using ReverTra Ace ® qPCR RT Kit (Toyobo, Osaka, Japan). Real-time PCR was used to measure the levels of mRNA for ovarian genes. Oligonucleotide primers corresponding to cDNA were designed using OMIGA 2.0 software (Oxford Molecular Ltd, Madison, WI, USA) (Table 1). The real-time PCR reactions were set up with SYBR Green PCR master mix (Toyobo) using a 7300 Real*-*Time PCR System (Applied Biosystems, Foster City, CA, USA), as described previously [28]; [31]. Relative mRNA levels were calculated using the 2^-ΔΔCT^ method and normalized to the endogenous reference gene ribosomal protein L32.

**Figure 6:**
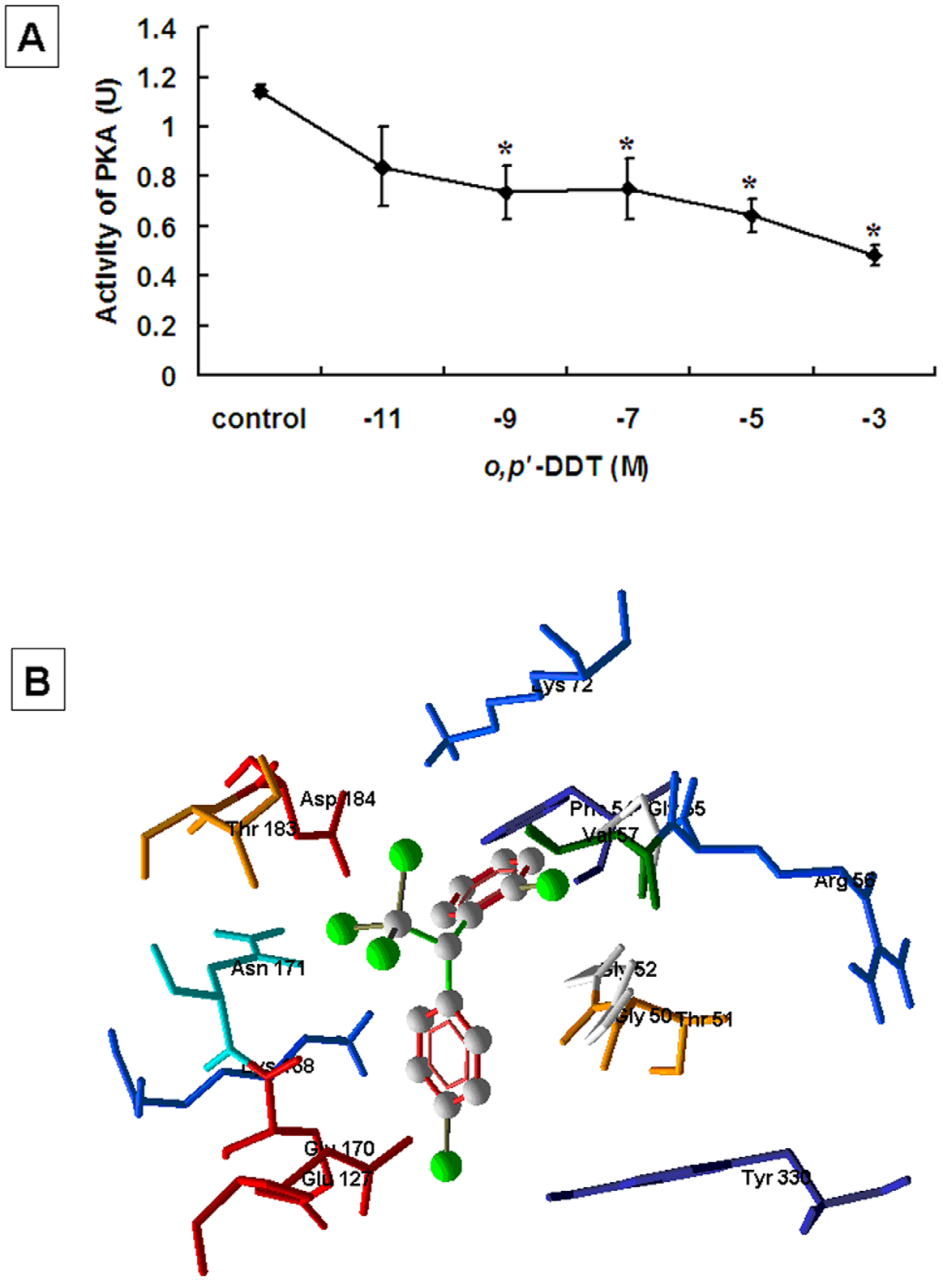
Direct inhibition of PKA activity by *o,p’*-DDT. (**A**) *o,p’*-DDT at concentration of 10^−11^ to 10^−3^ M was incubated with 1.25 U purified active catalytic subunit of PKA (control enzyme provided in the PKA PepTag Assay kit). PKA activity was measured using PepTag Assay. The kinase activity was quantitated, as described in *Materials and Methods*. The results of three independent experiments performed were shown as mean ± SEM. *, *ρ* <0.05, compared to control. (**B**) The interaction of *o,p’*-DDT with PKA. *o,p’*-DDT was represented in 3D with ball and stick models and the protein was represented in stick models.

### Measurement of Prostaglandin E2 (PGE2) in Cell Culture Media and Ovarian Tissue

Conditioned media was collected from granulosa cells exposed to *o,p’*-DDT at concentrations of 10^−12^ to 10^−8^ M for 6 h. Ovaries were collected from rats injected with *o,p’*-DDT as described above. The levels of PGE2 in conditioned media and ovarian tissues were measured using the EIA kits (Cayman Chemical Company, MI, USA) according to the manufacturer’s protocol. At the end of assay, the absorbance was read at 420 nm in the Infinite M200 plate reader (Tecan USA). The concentrations of PGE2 in samples were determined using the equations obtained from the standard curve plot.

### Generation of COX-2 Promoter-luciferase Reporter Plasmids and Luciferase Reporter Assay

A 384-bp (−364/+20) fragment of the rat COX-2 gene promoter was cloned into the pGL3 basic vector that is a firefly luciferase reporter plasmid (Promega, Madison, WI, USA) according to the methods described previously [29]; [30]. Granulosa cells were transfected with firefly luciferase reporter plasmids (pGL3-COX-2 promoter) and *Renilla* luciferase vector (pRL-TK vector) using a Lipofectamine 2000 reagent (Invitrogen). After 4 h, cells were exposed to *o,p’*-DDT at concentration of 10^−12^ to 10^−8^ M overnight. Firefly and *Renilla* luciferase activities were measured using a dual-luciferase reporter assay system (Promega) in the Infinite M200 plate reader (Tecan USA). Firefly luciferase activities were normalized by *Renilla* luciferase activities, and each experiment was performed in triplicate at least three times.

### Detection of Activity of PKA/PKC

Activity of PKA/PKC was measured using PepTag Assay according to the manufacturer’s instruction (Promega). For *in vitro* and *in vivo* experiments, endogenous PKA/PKC proteins were extracted from exposed cells or tissues. The cell lysate protein was incubated with PepTag PKA/PKC reaction buffer and PepTag A1/C1 peptide (as a PKA/PKC substrate). The reaction products were analyzed on 0.8% agarose gel and then photographed on a UV transilluminator. Phosphorylated PepTag A1/C1 peptide migrated toward the anode (+), while non-phosphorylated peptide toward the cathode (−). To test the direct inhibition of PKA activity, *o,p’*-DDT at concentration of 10^−11^ to 10^−3^ M was incubated with 1.25 U purified active catalytic subunit of PKA, substrate and reaction buffer. To quantify kinase activity, the negatively charged phosphorylated bands were excised from the gel and melted in 75 µl of Gel Solubilization Solution and 50 µl of glacial acetic acid. The absorbance was read at 570 nm in the Infinite M200 plate reader. The activity of PKA was calculated according to formula described in the manufacturer’s instruction (Promega).

### The Molecular Docking Method

The binding mode of *o,p’*-DDT to PKA was predicted by molecular docking method. The ligand was docked into the binding site of PKA by Molegro Virtual Docker (MVD) Version 4.2. The initial atomic coordinates of PKA were taken from the Protein Data Bank (PDB ID: 3FJQ). The binding pocket was defined as a sphere with a radius of 15 Å around the center of user-defined cavity. The grid resolution of 0.30 Å was used. MolDock SE optimizer was selected as the searching algorithm and energetic evaluations were evaluated with MolDock Score. For each molecular docking, 10 poses of the ligands were obtained from the molecular docking and the pose with the highest docking score was selected for the analysis.

### Statistical Analyses

All data are presented as means ± SEM. One-way ANOVA was used to test differences in MTS, levels of mRNA for each gene, PGE2 concentrations and PKA activities among treatments. If ANOVA revealed significant effects of treatments, the means were compared by Tukey’s test, with *ρ* <0.05 considered significant.

## Results

### Low Concentrations of *o,p’*-DDT Inhibit Gene Expression, PGE2 Secretion and PKA Activity in Rat Ovarian Granulosa Cells

To test whether low concentrations of *o,p’*-DDT are toxic to granulosa cells, cells were exposed to *o,p’*-DDT (10^−12^−10^−8^ M) for 24 h or 48 h and cell viability was determined. There were no significant changes in cell viability in *o,p’*-DDT-treated cells at all experimental concentrations (Fig 1).

To determine the effects of *o,p’*-DDT on the gene expression in granulosa cells, we examined the mRNA levels of genes that play important roles in ovarian functions. These genes include cytochrome P450 side chain cleavage enzyme (P450scc), steroidogenic acute regulatory protein (StAR), progesterone receptor (PR), estrogen sulfotransferase (SULT1E1), cyclooxygenase-2 (COX-2), epidermal growth factor epiregulin (EREG), runt-related transcription factor (RUNX1) and cell cycle regulator (p21). The real-time PCR data showed that the expression of these genes was inhibited by *o,p’*-DDT at relatively low environmental concentrations (10^−12^−10^−8^ M) (Fig. 2A). Notably, exposure to *o,p’*-DDT at picomole level (10^−11 ^M) decreased the mRNA levels of all genes examined in the experiments (Fig. 2A).

As prostaglandin E 2 (PGE2) is a key hormone for ovarian functions, we investigated the effects of *o,p’*-DDT on PGE2 secretion. As shown in Fig. 2B, exposure to *o,p’*-DDT at 10^−11^ M significantly decreased PGE2 production in the culture media of granulosa cells from 132.8±7.5 pg/ml to 93.1±2.6 pg/ml (*ρ*<0.05). The reduction of PGE2 level by *o,p’*-DDT reached a maximal of 71.3±1.5 pg/ml at concentration of 10^−8^ M. Because COX-2 is a rate-limiting enzyme in the biosynthesis of PGE2, we further examined whether *o,p’*-DDT affects COX-2 gene expression at transcriptional level. The results revealed that the luciferase activity of COX-2 promoter-reporter constructs was reduced in granulosa cells exposed to *o,p’*-DDT at 10^−11^−10^−8^ M compared with control cells (Fig. 2C).

It has been reported that PKA and PKC are critical signaling mediators in regulation of the expression of P450scc, StAR, PR, COX-2, EREG, RUNX1 and p21 genes [34]–[38].We determined PKA and PKC activities in granulosa cells exposed to *o,p’*-DDT (10^−12^−10^−8^ M). As shown in Fig. 3, the density of phosphorylated substrate peptide represents the activity of PKA or PKC. Exposure to *o,p’*-DDT at 10^−11^−10^−8^ M resulted in a marked inhibition of PKA activity (Fig. 3A), while the activity of PKC did not significantly changed in *o,p’*-DDT-treated cells (Fig. 3B).

### Low Doses of *o,p’*-DDT Affect the *in vivo* Expression of Genes, PGE2 Secretion and PKA Activity in Rat Ovaries

To determine whether *o,p’*-DDT had a similar effect on ovary *in vivo*, rats were daily administrated with 0.1, 0.5 and 1 mg/kg *o,p’*-DDT, and ovarian granulosa cells were collected for bioassays. A 6-day treatment with *o,p’*-DDT at doses of 0.5–1 mg/kg evidently decreased the expression levels of mRNA for PR, SULT1E1, COX-2, EREG, RUNX1 and p21 genes (Fig. 4A). A noticeable reduction of PR was observed in the rat ovaries exposed to the lowest dose of 0.1 mg/kg. The levels of PGE2 were decreased from 246.6±48.1 ng/ml to 133.0±11.4 ng/ml and 80.9±10.4 ng/ml in ovaries exposed to *o,p’*-DDT at 0.5 and 1 mg/kg, respectively (Fig. 4B). The activity of PKA was decreased by *o,p’*-DDT in a dose-dependent manner (Fig. 4C), while PKC activity was not significantly affected by this compound (Fig. 4D).

### Low Concentrations of *o,p’*-DDT Repress the Expression of Ovarian Genes and PKA Activity by ERs- and GPR30-independent Pathway

We further investigate whether *o,p’*-DDT disrupted the ovarian functions due to its estrogenic activity. E_2_ decreased the mRNA levels of ovarian genes in rat granulosa cells and the inhibitor of ERs (α and β), ICI 182780 reversed the inhibition of E_2_ (Fig. 5A). To test whether *o,p’*-DDT reduced the expression of ovarian genes and PKA activity by mimicking the action of E_2_ and binding to ERs, ICI 182780 was used to attenuate the pathways of ERs in the cells exposed to *o,p’*-DDT at low concentrations (10^−11^−10^−9^ M). Treatment with ICI 182780 alone had no effects on gene expression (Fig. 5B). The combined treatment with ICI 182780 and *o,p’*-DDT could not reverse the down-regulation of those genes and the decrease in PKA activity (Fig. 5B and 5D).

GPR30, a G protein-coupled receptor, has been recognized as the novel membrane ER in human and mice [39]. In the present study, the expression of GPR30 was identified in rat granulosa cells by RT-PCR (data not shown). We further examine whether *o,p’*-DDT repressed ovarian gene expression and PKA activity via GPR30 pathway. Gene expression had no changes in cells exposed to the specific antagonist of GRP30, G15 along (Fig. 5C). The combined treatment with G15 and *o,p’*-DDT did not block the inhibition of gene expression and PKA activation (Fig. 5C and 5D).

### Direct Inhibition of PKA Activity by *o,p’*-DDT

Because *o,p’*-DDT suppressed the activity of PKA extracted from granulosa cells by ERs- and GPR30-independent pathways, we further determined whether this inhibitory effect of *o,p'*-DDT is due to its direct interference with PKA. The 1.25 U purified active catalytic subunit of PKA was directly incubated with *o,p’*-DDT at concentrations of 10^−11^ to 10^−3^ M. The results indicated that PKA activity was repressed by *o,p’*-DDT in a dose-dependent manner (Fig. 6A). The activity of PKA was reduced by 35.8% in the samples incubated with *o,p’*-DDT at 10^−9^ M (Fig. 6A). Molecular modelling study further revealed the direct interaction of *o,p’*-DDT with PKA catalytic subunit protein. *o,p’*-DDT fits well at the ATP-binding site of PKA (Fig. 6B).

## Discussion

In this study, we demonstrated for the first time that very low doses of *o,p’*-DDT (picomolar or nanomolar) suppressed the expression of ovarian genes and production of PGE2 via inhibiting the activation of PKA signaling pathway. The low concentrations of *o,p’*-DDT that are able to produce such an effect both *in vitro* and *in vivo* give this observation environmental relevance. An important and surprising conclusion from our studies was that this inhibitory effect was exerted independently of either classical ERs or GPR30. Instead, our data suggested that *o,p’*-DDT directly interfere with the activity of the PKA catalytic subunit. Our novel findings support the hypothesis that EDCs such as *o,p’*-DDT regulate gene expression through multiple elements beyond receptor binding.

PGE2, an important hormone produced by ovarian granulosa cells, is one member of prostaglandins (PGs). PGs are ubiquitous tissue hormones and play pivotal roles in reproduction, immunity and inflammation [40]; [41]. But very few studies have examined the action of EDCs on the synthesis of this family of hormones. It has been reported that eggshell thinning induced by *p,p’*-DDE in avian species was accompanied by reduced activity of prostaglandin synthetase, reduced levels of PGE2 [42]. Our previous studies demonstrated that bifenthrin, a widely used pesticide, inhibited PGE2 production in rat ovarian cells [28]; [31]. A recent study showed that many known EDCs at concentrations of 10^−7^−10^−4^ M inhibited the PG pathway in human primary mast cells [43]. In the present study, *in vitro* exposure to *o,p’*-DDT at a concentration as low as 10^−11 ^M significantly attenuated the production of PGE2 in granulosa cells. *In vivo* experiments consistently showed that *o,p’*-DDT at 0.5–1 mg/kg inhibited PGE2 levels in ovarian tissues. The true rate-limiting step in PG biosynthesis is believed to be under the control of the COX-2 enzyme. This study showed that *o,p’*-DDT suppressed the expression of COX-2 gene at the transcriptional level. Collectively, these *in* *vitro* and *in vivo* observations suggest that PGs inhibition as an important end point should be taken into consideration in the risk assessment of EDCs such as *o,p’*-DDT at low environmental doses.

In the present study, exposure to *o,p’*-DDT at picomolar or nanomolar significantly inhibited the *in vitro* expression of ovarian specific genes. These results are consistent with the *in vivo* observation that most of these genes are decreased by treatment with *o,p’*-DDT at dose of 0.5–1 mg/kg. These genes are involved in multiple ovarian functions, including progesterone synthesis (P450scc and StAR), estrogen metabolism (SULT1E1), prostaglandins biosynthesis (COX-2), gene transcription (PR and RUNX1), granulosa cell proliferation (EREG), and granulosa cell differentiation (p21) [29]; [44]–[47]. Given the cross-communication signaling pathways of these genes in the regulation of ovarian functions, our results indicate that *o,p’*-DDT at environmental relevant concentrations may disrupt the network of ovarian gene expression profiles.

The evidences that E_2_, synthetic estrogens and some xenoestrogens suppressed ovarian processes such as progesterone production and P450scc expression in granulosa cells [26]; [48]; [49], lead us to investigate whether *o,p’*-DDT disturbs the ovarian gene expression due to its estrogenic activity. In this study, the effects of *o,p’*-DDT on decrease in the expression of ovarian genes, are similar to that of E_2_. However, our data showed that ICI 182780 could not prevent the inhibitory action of *o,p’*-DDT on gene expression in rat ovarian cells, indicating a non-ER (α and β) mechanism. In agreement with our data, a recent study showed that ICI 182780 did not affect the inductive effects of *o,p’*-DDT on aromatase expression in human breast cancer cells [50]. Frigo et al. demonstrated that DDT-related compounds altered gene expression via an ER-independent pathway in human uterine cells [51]. Kiyosawa et al. found that *o,p’*-DDT-elicited hepatic gene expression is not mediated by the ER but rather through PXR/CAR-dependent mechanisms [52]. The ER-independent mechanism was also observed in the actions of other xenoestrogens. For example, methoxychlor could stimulate estrogen-responsive uterine genes, lactoferrin and glucose-6-phosphate dehydrogenase, in the uterus of ERα-knockout mice despite the addition of ICI 182780 [53]. Exposure to bisphenol A caused neuronal cell death through an ER-independent neurotoxic mechanism [54]. These previous observations, along with our present findings, strongly suggest that xenoestrogens, as shown here for *o,p’*-DDT, could exert disrupting effects through multiple pathways rather than classical ERs.

The newly described membrane ER, GPR30, provides explanations for the more potent actions of some xenoestrogens independent of the classical ERs [55]; [56]. For instance, low doses of bisphenol A promote human seminoma cell proliferation via GPR30 but not classical ERs [57]. However, our present data showed that the inhibition of gene expression by *o,p’*-DDT was not reversed by GPR30 antagonist. Additionally, *o,p’*-DDT has ineffective binding affinity for human GPR30 [56]. These observations indicate the actions of *o,p’*-DDT in a GPR30-independent manner. Neither classical ERs nor GPR30 mediated the inhibitory effects of *o,p’*-DDT, implicating that this compound may modulate ovarian gene expression through other intracellular signaling events.

It has been well established that PKA and PKC are critical mediators of the signaling transduction pathway to control the expression of ovarian genes examined in this study [34]–[38]. In this study, we found that both *in vitro* and *in vivo* exposure to *o,p’*-DDT decreased the activity of cellular PKA, but not PKC. Importantly, *o,p’*-DDT could directly inhibit the activation of purified catalytic subunit of PKA. This observation was confirmed by our molecular docking study that *o,p’*-DDT competes with ATP and occupies the ATP-binding site of PKA, which hinders the binding of ATP to PKA catalytic region, consequently resulting in the inhibition of PKA activity. Taken together, these data suggest that the disruption of gene expression by *o,p’*-DDT occurs through direct interference with the activity of the PKA catalytic subunit, rather than binding to classical ERs or GPR30. Considering that PKA is an effector to trigger a signaling cascade and amplify the intracellular response, this would help explain the results demonstrating the ability of *o,p’*-DDT at very low doses to significantly decrease ovarian gene expression similar to E_2_, despite an much weaker affinity to ERs and GPR30 than E_2_. Therefore, future studies must consider a mechanism by which EDCs alter gene expression or hormone action not by receptor binding but rather via modification in the signaling mediators, such as direct interference with kinase proteins, which may answer why some EDCs have large effects at small doses despite of lower affinity to receptors than natural hormones [58].

About 25% of infertility cases in women are due to problems with ovarian functions [59]. The adverse effects of EDCs on female reproduction have been linked to ovarian dysfunctions [60]. Studies have shown that exposure of female rats and mice to *o,p’*-DDT at high doses results in acceleration of the loss of fertility, referred to as ovarian dysfunction [23]; [24]. Given its potent inhibition in gene expression and prostaglandin synthesis, the exposure of women to *o,p’*-DDT at low concentrations may exert deleterious effects to ovarian functions. It is worth noting that in the present study *o,p’*-DDT causes changes in ovarian cell functions at concentrations between 10^−11^ M and 10^−8^ M. Unfortunately, the mean and median range of *o,p’*-DDT in women blood and other tissues that found to be from 0.3 ng/g to 500 ng/g (8.46×10^−10^ M−1.41×10^−6^ M) [3]; [18]–[22], exceeds the concentrations used in the present experiments. It implies that the current exposure levels of *o,p’*-DDT observed in the population likely poses a health risk to female reproduction.

In conclusion, exposure to *o,p’*-DDT at environmental relevant concentrations, may interfere with signaling kinase PKA, consequently results in alteration in ovarian gene expression and PG synthesis, and then leads to disruptions in ovarian processes, which may link to the frequent adverse outcomes of women reproduction with an increasing incidence. Thus, the risks to human health of this environmental contaminant should continue to be cautiously and carefully considered.
